# Removal of microplastics from aqueous media using activated jute stick charcoal

**DOI:** 10.1016/j.heliyon.2024.e37380

**Published:** 2024-09-03

**Authors:** Nur Alom, Tapati Roy, Tanny Sarkar, Md Rasel, Md Sanwar Hossain, Mamun Jamal

**Affiliations:** a Department of Chemistry, Khulna University of Engineering & Technology, Khulna, 9203, Bangladesh; b Department of Agronomy, Faculty of Agriculture, Khulna Agricultural University, Khulna, Bangladesh; c Microplastics Solution Ltd., Incubation Centre, KUET Business Park, Khulna, Bangladesh

**Keywords:** Adsorption, Density functional theory, Kinetics, Isotherm and PVC microplastics

## Abstract

Microplastics (MPs), which are repositories of various pollutants, have significant effects on the people and the environment. Therefore, there is an urgent need for efficient and eco-friendly techniques to eliminate microplastics from water-based environments. This study introduces a new method for producing jute stick-activated charcoal (JSAC) by placing jute sticks on high-temperature pyrolysis without oxygen, followed by chemical activation with HCl. This process greatly enhances the adsorption capacity of JSAC for polyvinylchloride-based microplastics (PVC-MPs). JSAC was characterized using UV–Vis, FT-IR, XRD, and SEM studies both before and after adsorption. The study investigated the influence of pH, adsorbent quantity, and contact time on the optimization of the JSAC process. The PVC-MPs exhibited a maximum adsorption capacity of 94.12 % for the target MPs (5 g L^−1^) within 120 min when 10 g L^−1^ of JSAC was added at pH 7.

This work also examined adsorption rate and various isotherm models. Adsorption kinetics analysis reveals electrostatic, hydrogen bond, π-π, and hydrophobic interactions are the combined forces responsible for MPs adsorption onto JSAC. However, the decrease in hydrophobicity in acidic or basic media led to a decrease in adsorption. The isotherm analysis was conducted using the Langmuir isotherm model, and predicted the maximum adsorption capacity of PVC-MPs to be 4.4668 mg/g. Furthermore, by employing density functional theory, the interaction energy after PVC-MP adsorption was calculated to be −269 kcal/mol, demonstrating robust adsorption and agreement with the experimental findings. Due to its large surface area and porous structure containing many functional groups, JSAC can potentially be used to treat MP contamination in water.

## Highlights

• An approach for removing MPs using jute stick activated charcoal (JSAC) was proposed.
• Removal efficiency of 94.12 % for MPs was found using JSAC.
• Electrostatic, hydrogen bonds, hydrophobic, and π-π interactions were the main adsorption forces.
• The experimental data is well-fitted with the Langmuir adsorption isotherm and pseudo-first-order kinetic model.

## 1 Introduction

Plastic pollution is a worldwide problem that poses a significant threat to the environment and has the potential to harm human health [1]. Marine ecosystems discover almost 12 million metric tons of plastic every year. According to projections, if the current patterns of plastic manufacture and consumption continue, by 2050, the amount of plastic in the oceans will exceed the population of fish [[2], [3], [4]]. Microplastics are chemically stable and do not pose a direct threat to human health. However, conclusive determination of their impact on human well-being remains elusive [5]. However, studies have confirmed that plastic pollution causes harm to aquatic organisms [6,7]. The release of additives from plastics during degradation produces significant detrimental byproducts. Researchers have extensively studied the characterization of "macroparticles," a crucial issue in understanding the breakdown of plastics into microplastics (MPs). Examples of MPs, commonly discussed, include Bisphenol A (BPA) and phthalates, used as plasticizers in PVC [8].

In addition, MPs provide a surface area that can adsorb different contaminants [9,10]. This feature necessitates a thorough analysis before we can conclusively confirm or reject the probability. It is possible to imagine that microparticles of a specific mass have a greater surface area than macroparticles of the same mass. Joo et al. examined the adsorption mechanisms of contaminants, namely per-/polyfluoroalkyl compounds. They identified three key processes: electrostatic repulsion, electrostatic interaction, and hydrophobic contact [11]. Polycyclic aromatic hydrocarbons (PAHs), which attract organic pollutants, pharmaceutical residues, insecticides, and heavy metals, pose a significant threat to aquatic life [12].

Microplastics (MPs), particles with a diameter of <5 mm, have been a major focus of concern in plastic pollution [13,14]. The long-lasting presence of MPs in natural environments can be due to their strong anti-corrosion properties [15,16]. As a result, MPs have the ability to consistently absorb persistent organic contaminants, heavy metals, and viruses from their surroundings. This poses a potential danger to the organisms that consume them as well as to the overall ecosystem, as contamination can spread via the food chain [[17], [18], [19]]. Anthropogenic wastes, such as sewage, attract MPs []. Despite the fact that sewage treatment facilities (STPs) effectively removed 90 % of MPs due to the significant volume of wastewater they released, these MPs still found their way into natural ecosystems in substantial quantities. STPs release a daily range of 15,000–4.5 million microplastic particles into surface waters; however, the rate of clearance may vary. Nanohybrid-based advanced oxidation processes (AOPs) are commonly used to eliminate MPs from water [20]. Activated peroxymonosulfate creates very reactive radicals in this method, which breaks down MPs [21].

On the other hand, the AOP requires precise operational conditions, and the intermediate substances produced during the breakdown of MPs can cause additional pollution in water environments. Membrane disc filters are an excellent method for removing MPs, successfully separating around 79.4 % of these particles [[22], [23], [24]]. However, obstruction of filter modules can easily occur, leading to an increase in operational costs. Filtration encounters obstacles, such as decreased effectiveness caused by fouling and the risk of pollution from improperly handled waste membranes. Granular activated carbon and amino-functionalized zeolites effectively adsorb polyethylene-MPs and polystyrene-MPs, respectively [25,26]. The authors mentioned that both adsorbents are highly effective at removing MPs, owing to the involvement of chemisorption, physisorption, hydrophobic, and electrostatic interactions [26,27]. However, the use of effective adsorbent is limited due to some disadvantages, such as the requirement for significant modification of the adsorbent in order to remove MPs effectively. Jute stick activated charcoal (JSAC) is a new adsorbent material that has high potential to reduce pollutants. JSAC's hydrophobic and organophilic qualities make it ideal for organic pollutant adsorption in water purification and solvent recovery systems [24,28]. Just to mention, carbonaceous materials' non-polar surfaces make JSAC hydrophobic. Activation by physical or chemical means removing many surface functional groups, especially those that contain oxygen. This leaves behind a carbon-rich, less polar surface. These charcoals have multiple adsorption sites and large, specific surfaces. Activated charcoal has polar and nonpolar basal sites in oxygen-containing functional groups [29], and hydrophobic JSAC binds nonpolar MPs, such as PVC [24]. Jute sticks are available as cheap agricultural byproducts, with a huge surface area and a good number of functional groups, and shows greater adsorption capacity [24,25,30,31].

Thereby, in this work we aim to use JSAC to remove PVC-MPs from an aqueous medium and investigate its mechanism. These findings should illuminate MP removal technology. We simultaneously investigated the effect of pH, adsorbent amount, and contact time on MP degradation. We also examined the adsorption kinetics, isotherms, optimum geometry, and interaction energy, which are crucial to understanding the adsorbent-adsorbate interaction. According to our understanding, JSAC's use of aqueous media for treating PVC-MPs is a pioneering step in this field.

## 2 Materials and methods

### 2.1 Materials

The jute sticks were collected from the local market in Khulna. PVC-MPs were purchased from BDH in England. Hydrochloric acid and sodium hydroxide were purchased from Merck, Germany. The experiments were conducted using ultrapure Millipore water (∼18.2 MΩ cm), and all reagents were laboratory-grade and used without further purification.

### 2.2 Preparation of jute stick activated charcoal (JSAC)

The jute sticks were chopped into small pieces and washed with water, followed by deionized water (DIW). The sticks were cleaned and dried in an oven at 110 °C for 24 h. A home blender was used to powder the dry sticks. The powder was placed in the furnace and heated for 1 h at 300, 400, and 500 °C [32,33]. During the pyrolysis process, jute stick powder was heated in an oxygen-free environment. This was achieved by conducting the pyrolysis in a controlled furnace with an inert atmosphere, using a continuous flow of nitrogen gas. The inert atmosphere prevents aerial oxidation and ensures that the organic material undergoes thermal decomposition rather than combustion [34] (Supplementary S1, Table S1). After being washed twice with DIW, the charcoal was activated by 0.5 M HCl. Briefly, a 10 % hydrochloric acid solution was prepared. Subsequently, charcoal powder was soaked in the hydrochloric acid solution, maintaining a ratio of 1:10. The soaked charcoal sample was left at room temperature for 6 h. Following this, the soaked sample was rinsed with distilled water to remove any excess acid solution, and the pH level was monitored to ensure neutrality. The sample was then filtered using a vacuum dryer and dried in an oven at a temperature of 80 °C [35].

### 2.3 Removal of MPs experiments

The removal of MPs was tested by adding 5g.L^−1^ PVC-based MPs and 10 mL DI water to a 50 mL conical flask, along with varying doses of JSAC. All of the MPs added conical flasks were shaken for 120 min at 150 rpm and 25 °C using a mechanical shaker. Thus, JSAC was used to separate the MPs from the aqueous solution [36].

### 2.4 Adsorption

The kinetics of JSAC adsorption were studied by filling a 50 mL conical flask with MPs, JSAC, and DIW, shaking at 150 rpm for 120 min. Utilizing the results obtained from the kinetic experiments, we calculated the adsorption equilibrium time. Subsequently, this equilibrium time was employed to establish the adsorption isotherms under the conditions of 10 gL^-1^ JSAC, 10 mL solution, and varying doses of 1–5 gL^-1^ of MPs particles. Using adsorption isotherm studies, the effects of pH on MPs adsorption by JSAC were examined.

The quantity of MPs adsorbed by AJSC, expressed in mg.g^−1^, was determined using a mass balance equation:

(1) $$Qe=\frac{\left(\mathrm{Co}-\mathrm{Ce}\right)\;V}{m}$$

(2) $$R=\frac{\left(\mathrm{Co}-\mathrm{Ce}\right)}{\mathrm{Co}}\times 100\%$$

here, C_0_ (mg/L) is the initial concentration, C_e_ (mg/L) is the concentration at the equilibrium of adsorption, V (L) is the volume of the solution, and m (g) is the quantity of the adsorbent.

### 2.5 Characterization of MPs and JSAC

Several methods were used to look at the samples' physical and chemical properties. These included an X-ray diffractometer (D2 PHASER, BENCHTOP XRD), a scanning electron microscope (JCM-7000, BENCHTOP SEM), fourier transform infrared spectroscopy (FTIR, IR Tracer 100, SHIMADZU), and a UV–Vis spectrophotometer (UV 1800, SHIMADZU). FTIR spectroscopy was employed to obtain spectra for the samples within the range of 500–4000 cm^−1^ at a resolution of 0.25 cm^−1^. The KBr pellet method was used for FTIR measurements, with 50 scans per spectrum, and pH values were measured using a pH meter (HANNA pH Tester).

### 2.6 Computational method

Computational calculations were conducted using the Gaussian 9, Revision C.01 series of programs [37]. The energy of geometries, Mulliken charge, and infrared (IR) properties were computed utilizing the DFT-PBEPBE/6-311+G (d, p) level of theory. The structure of JSAC and PVC-MPs underwent complete optimization using the DFT-B3LYP/6-311+G (d,p) method simultaneously.

## 3 Results and discussion

### 3.1 Scanning electron microscopy (SEM)

**Fig. 1:**
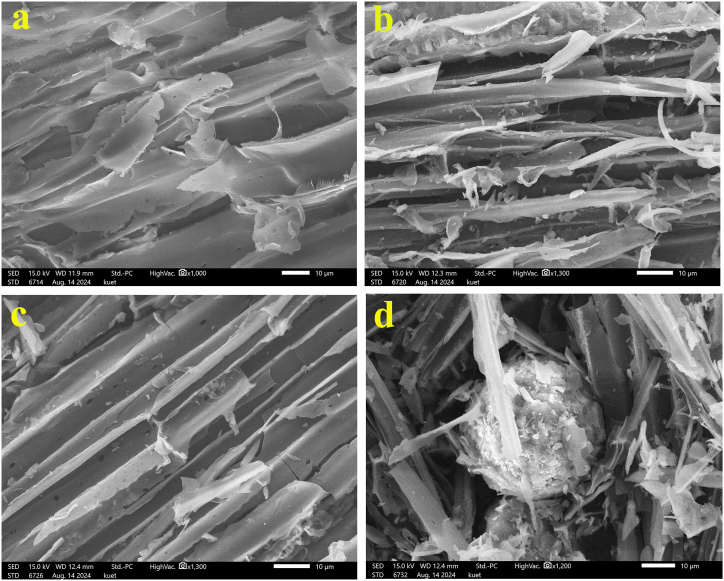
Scanning electron micrograph of (a) JS powder; (b) JSC; (c) JSAC; (d) JSAC after adsorption.

Fig. 1 shows the SEM images of (a) JS powder (JS) (b) jute sticks charcoal (JSC), (c) jute sticks activated charcoal (JSAC), and (d) JSAC after adsorption. The images depict the morphological changes of carbon materials during pyrolysis, activation, and adsorption. The jute stick surfaces are relatively smooth solid surfaces with long ridges (Fig. 1a), resembling a series of parallel lines as observed in other biomasses [38]. Jute stick pyrolysis produces charcoal with organic volatiles, leaving a ruptured surface with small pores (Fig. 1b). Asadullah et al. found similar results with a composition of 49.79 wt% C, 6.02 wt% H, 41.37 wt% O, 0.19 wt% N, 0.05 wt% Cl, and 0.05 wt% S [39]. Fig. 1c illustrates the activation of charcoal with HCl, showcasing various pores with increased surface area. The number of pores in the case of JSAC is higher than that of JSC. The adsorption efficiency of JSAC is confirmed by the visible presence of PVC-MPs (Fig. 1d), indicating lignin dissolution from jute sticks through HCl activity. (Supplementary S2, Fig. S1).

### 3.2 FTIR of JSC before & after activation and adsorption

**Fig. 2:**
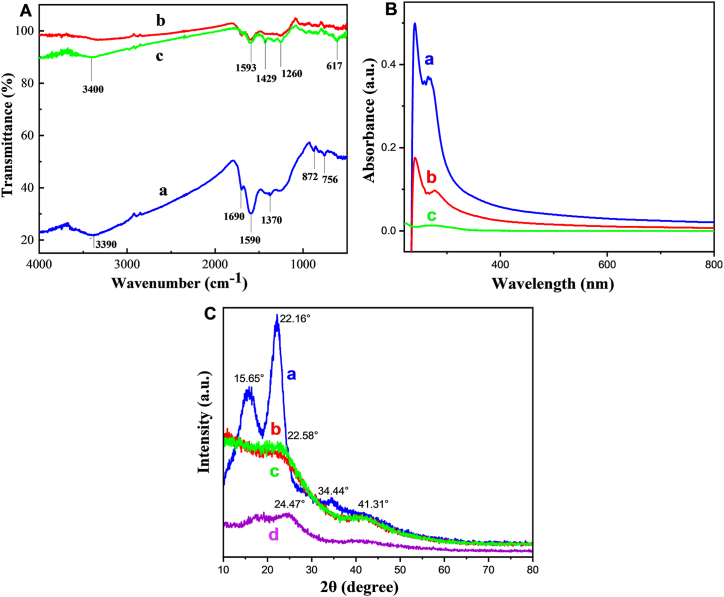
A. FT-IR analysis of JSC; (a) before activation; (b) after activation; (c) JSAC after adsorption; Fig. 2B. UV–Vis spectra of the PVC-MPs mixture (a); the PVC-MPs filtered through paper (b); and the PVC-MPs removed using JSAC (c); and Fig. 2C. XRD of jute sticks powder; JSC; JSAC; and JSAC after adsorption.

Due to the existence of oxygen-containing functional groups, activated carbon possesses both polar and non-polar basal sites [27]. Fig. 2A depicts the FT-IR analysis of JSC. Spectrum (a) represents the analysis before activation, (b) represents the analysis after activation, and (c) represents the analysis of JSAC after adsorption. The distinct peak found at 3390 cm^−1^ in Fig. 2A (a) corresponds to the O-H stretching vibration of cellulose, pectin, absorbed water, hemicellulose, and lignin [33,40]. Just to mention, jute sticks contain a variety of phytochemicals, including cellulose, hemicellulose, lignin, and other minor components. These compounds contribute to the presence of functional groups such as hydroxyl, carbonyl, and carboxyl groups in the raw material. During the pyrolysis process at 500 °C, carbonyl functional groups present in the raw material undergo significant transformations. Carbonyl groups from cellulose, hemicellulose, and lignin decompose, leading to the formation of various volatile compounds and a carbonaceous solid residue. The carbonization process results in the formation of more stable aromatic structures. The unique peak observed at 3390 cm^−1^ in Fig. 2A (a), corresponds to the O-H stretching vibration of cellulose, pectin, absorbed water, hemicellulose, and lignin [9]. Additionally, the surfaces of JSC, JSAC, and JSAC after adsorption may adsorb moisture from the environment during handling and storage prior to FTIR analysis.

The carbonyl group (C=O) was shown to be responsible for the strong band at 1690 cm^−1^. The aromatic C=C bonds were assigned a band at 1590 cm^−1^, whereas the aromatic methyl group was assigned a band at 1370 cm^−1^ (CH_3_). The 872 and 756 cm^−1^ bands correspond to = CH_2_ groups and C–H bending vibration, respectively [41]. The FTIR spectra of Fig. 2A (b) showed different functional groups due to the activation of JSC. It's presented that the C=O stretching of the carbonyl group in the quinone molecule was responsible for the prominent peak at 1585 cm^−1^. In aromatic groups, the 1429 cm^−1^ band showed C–O stretching [39]. The FTIR spectra of JSC shown in Fig. 2A (c), with various functional groups formed during adsorption by activated JSC. The bands at 1429 and 617 cm^−1^ were related to (C=C) in aromatic groups and Cl^−^, respectively.

### 3.3 UV–Vis spectroscopy analysis

Fig. 2B illustrates the UV–Vis spectra of the PVC-MPs mixture (a), the PVC-MPs filtered through paper (b), and the PVC-MPs removed using JSAC (c). Fig. 2B demonstrates that the absorptive intensity of all PVC-MPs diminishes progressively when JSAC is added to the PVC-MPs solution. This outcome illustrates that JSAC possesses a greater number of active sites on its surface, hence augmenting the process of adsorption. The concentrations of PVC-MPs prior to and after adsorption were assessed by measuring the absorptive intensities at wavelengths of 240 and 297 nm. Following the application of JSAC, there was a notable decrease in absorbance at these specific wavelengths, suggesting a substantial adsorption of MPs by JSAC [42,43].

### 3.4 X-ray diffraction (XRD)

Fig. 2C presents the XRD spectra of jute stick (JS) powder (a), jute stick charcoal (JSC) (b), jute stick activated charcoal (JSAC) (c), and JSAC after microplastics (MPs) adsorption (d). The XRD pattern of jute stick powder (Fig. 2C; a) displays broad diffraction peaks, indicative of its predominantly amorphous structure. This finding aligns with the lignocellulosic composition of jute, which consists mainly of cellulose, hemicellulose, and lignin [33]. The prominent peaks detected at around 2θ = 15.65°, 22.16°, and 34.44° are indicative of the crystalline arrangement of cellulose, which is commonly found in natural plant fibers. The presence of peaks corresponding to the (110) and (200) planes of cellulose confirms that the cellulose in the jute sticks has a semi-crystalline structure.

For JSC, the diffraction peak at 2θ = 22.58° is attributed to the (002) plane of graphitic carbon, a characteristic of amorphous carbon structures with limited crystallinity (Fig. 2C; b). The presence of a peak at 2θ = 41.31° corresponds to the (100) plane, associated with graphitic crystallites. Although this peak is less intense, it further verifies the existence of graphitic domains within the predominantly amorphous carbon matrix [33]. The emergence of this peak suggests that partial graphitization occurred during the carbonization process, resulting in the formation of small, ordered graphitic regions within the otherwise amorphous structure.

Following the activation of JSC, the XRD pattern (Fig. 2C; c) exhibits two broad peaks around 2θ = 21° and 41°, corresponding to the carbon (002) and (101) planes, respectively. These peaks can be attributed to the porous graphitic framework [24]. The XRD analysis indicates that a graphitic structure was formed after the activation process. However, the broadening of these peaks suggests a low degree of graphitization, characterized by small domains of coherent and parallel stacking of graphene layers.

After MPs adsorption, the XRD pattern shows a noticeable shift in the (002) peak to 2θ = 24.47°, commonly associated with graphitic carbon structures (Fig. 2C; d). This shift from the previously observed peak at 21° in the XRD pattern of JSAC before adsorption suggests enhanced ordering within the carbon structure. The peak at 2θ = 41° remains unchanged, indicating the persistence of graphitic domains within JSAC. Overall, the XRD pattern of JSAC exhibited minimal changes after being used for MPs removal, indicating the material's structural stability during the adsorption process [30].

### 3.5 Effect of pH on the adsorption

**Fig. 3:**
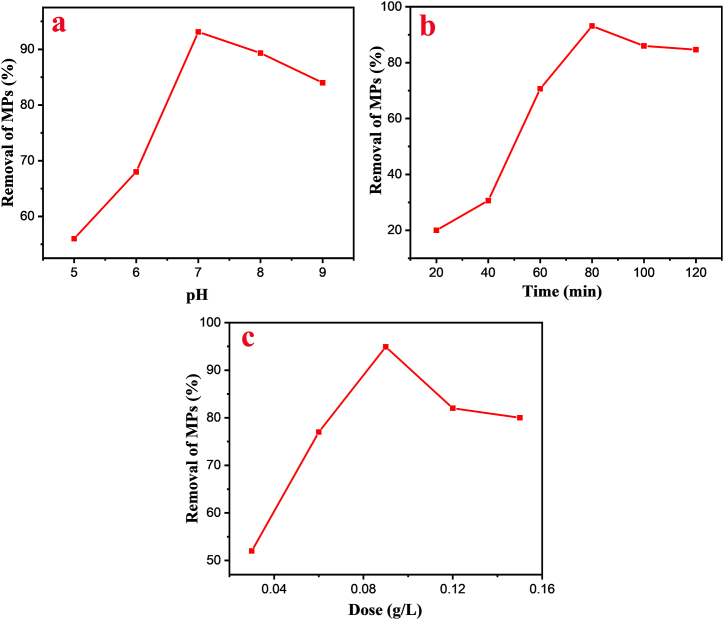
Effect of (a) pH on the adsorption; (b) contact time for MPs removal; (c) dose for MPs removal.

The pH of a solution significantly influences the adsorption process in experiments, as it is directly linked to the structure of both contaminants and adsorbents. Thereby, optimization was conducted to achieve maximum adsorption of PVC-MPs by JSAC across pH ranges 5–9, using 0.075 and 0.15 g/mL, respectively (Fig. 3a). Microplastics showed the highest adsorption capacity for JSAC in neutral solutions, while in acidic solutions, JSAC adsorbs fewer MPs to reach equilibrium. The reduced adsorption capacity under acidic conditions may be attributed to the formation of acidic oxygen-containing groups on the JSAC surface. This led to a reduction in hydrophobicity and hindered the hydrophobic interaction between PVC-MPs and JSAC. As a result, this influenced the hydrophobic interaction between PVC-MPs and JSAC [44]. Hence, the most effective adsorption of JSAC by MPs occurred under neutral conditions, with MPs exhibiting a reduced influence in alkaline conditions compared to acidic conditions.

### 3.6 Effect of contact time

Adsorption occurs in three stages: rapid adsorption, lower adsorption than initial, and equilibrium state. Rapid adsorption occurs due to mass transfer, while reduced adsorption is due to minimal peripheral active binding sites (Fig. 3b). Finally, the availability of active sites for continued adsorption has become few, resulting in the formation of the adsorption equilibrium stage [45]. Here, maximum adsorption (about 94.12 %) was observed at a duration of 80 min. It is evident that there was a swift uptake as the contact time expanded, followed by a gradual decline until reaching equilibrium at approximately 80 min. This phenomenon can be explained by considering the electrostatic repulsion between MPs and JSAC, the hydrophilic-hydrophobic interactions between MPs and JSAC, and the reduced adsorption behavior of MPs on the JSAC surface, which collectively hinder the rapid decrease in removal efficiency [46].

### 3.7 Effect of adsorbent dose

Adsorbent dosage is a critical parameter that influences the amount of adsorbed substrate. The removal efficiency of MPs improved in tandem with the increase in JSAC dose. The inclusion of adsorbents increases the overall surface area. However, process modification is required to minimize excessive adsorbent consumption. Because active binding sites are increasingly accessible which improves the efficacy of MPs removal steadily with the rise of adsorbent quantity. The removal rate remains constant even after adding an extra amount in the equilibrium state due to the introduction of more active sites compared to lower adsorbate concentrations [47]. In this study, different doses of JSC (2 = 0.03, 4 = 0.06, 6 = 0.09, 8 = 0.12, and 10 = 0.15 g/L) were selected to determine the removal efficiency. Fig. 3c shows a significant increase in adsorbent amount from 0.03 to 0.09 g/L, followed by a gradual decrease. Due to an excessive concentration of JSAC adhering to the MPs surface, surface flaws were induced. Hence, considering its exceptional rejection capacity and high-water permeability, JSAC concentration of 6 g/L was deemed optimal for the experiment.

### 3.8 Adsorption kinetics

**Fig. 4:**
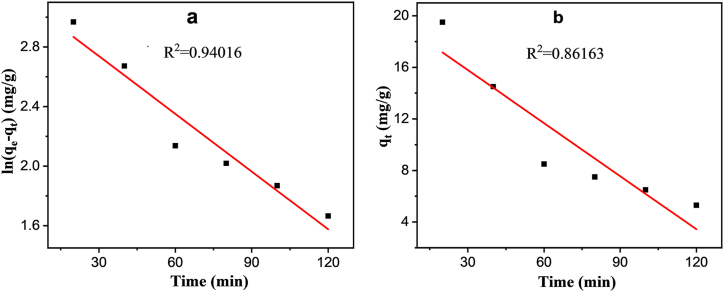
Linear Plot of (a) pseudo-first-order and (b) pseudo-second-order kinetic model.

In the realm of adsorption, kinetics plays a pivotal role by influencing mechanisms such as mass transfer, reagent reaction, and diffusion control. The adsorption process unfolds in three distinct phases: the diffusion of adsorbate up to the boundary layer, the diffusion of adsorbate from the surface to the internal sites of the adsorbent, and the migration of adsorbate on the surface of the adsorbent. To assess the adsorption characteristics, the pseudo-first-order model (Fig. 4a) was employed for determining the adsorption rate, while the pseudo-second-order model (Fig. 4b) was utilized to unravel the nature of adsorption.

The adsorption mechanism's suitability is assessed using coefficient value (R^2^) [48,49]. The study investigated the effect of contact time on the adsorption of MPs by JSAC by adding 5 gL^-1^ MPs and 10 gL^-1^ JSAC to the testing bottle. The adsorption procedure's kinetics were analyzed using pseudo-first-order and pseudo-second-order kinetics models, determining the amount of JSAC adsorbed at a specific time 't'.

#### 3.8.1 Pseudo-first-order kinetics model

The pseudo-first-order kinetics is presented as

$$\ln\left(q_{e}-q_{t}\right)=\ln\;q_{e}-k_{1}t$$

Where ${\;q}_{t}$ (mg g^−1^) and $q_{e}$ (mg g^−1^) symbolize the amounts of the JSAC adsorbed at any time t (min) and equilibrium, $k_{1}$ (min^−1^) is the first-order rate constants. The pseudo-first-order kinetics model focuses on the physisorption of pollutants, with the adsorption process primarily influenced by the nature of the adsorbate [30,49].

#### 3.8.2 Pseudo-second-order kinetics model

The pseudo-second-order kinetics model assumes that the rate-limiting step involves electron transfer between the adsorbate and adsorbent.

The kinetic model is articulated as follows:

$$\frac{t}{q_{t}}=\frac{1}{k_{2}q_{e}^{2}}+\frac{t}{q_{e}}$$

Where ${\;q}_{t}$ (mg g^−1^) and $q_{e}$ (mg g^−1^) represent the amounts of the JSAC adsorbed at any time t (min) and at equilibrium respectively, $k_{2}$ (min^−1^) is the second-order rate constants. Here, the chemisorption of the pollutants is determined by the pseudo-second-order kinetics model [30]. The parameters obtained from the kinetic models are presented in Table 1.

**Table 1:** Parameters derived from the kinetics model.

Pseudo First order			Pseudo Second Order		
q_e_ (mg/g)	K_1_ (min^−1^)	R^2^	q_e_(mg/g)	K_2_ (min^−1^g.mg^−1^)	R^2^
22.83	0.0129	0.94	7.14	0.000985	0.86

In this study, Fig. 4a represents pseudo-first-order and Fig. 4b represents pseudo-second-order kinetics model where the R^2^ value of first-order and second-order is 0.94016 and 0.86163, respectively. Here, the value of R^2^ is higher in the first-order than that of the second-order kinetics model. Hence, it is evident that the adsorption of PVC-MPs by JSAC can be effectively characterized using the pseudo-first-order model. Thus, it can be concluded that the physisorption occurred with irreversible adsorption between PVC-MPs and JSAC, and there was chemosorption due to the availability of functional groups.

### 3.9 Adsorption isotherm

**Fig. 5:**
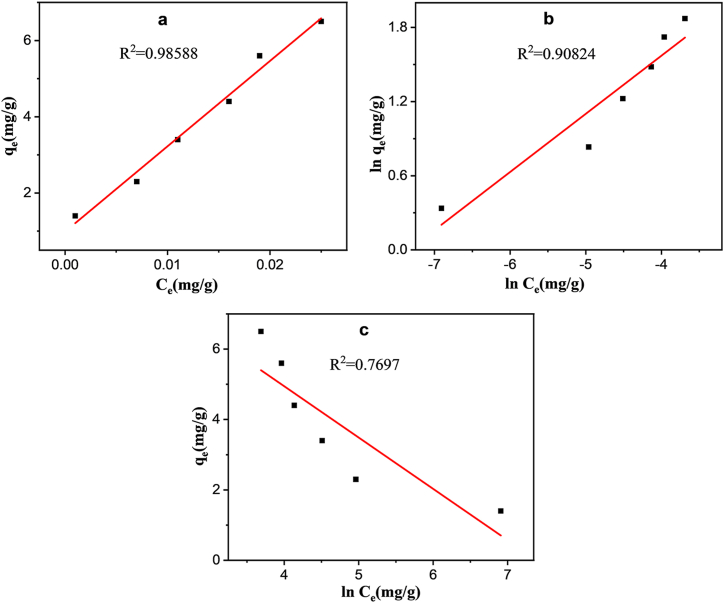
Linear plot of (a) Langmuir isotherm; (b) Freundlich isotherm; (c) Temkin isotherm.

Adsorption isotherm analysis is crucial for understanding the interaction between adsorbate and adsorbent, thereby assessing the efficacy of adsorption studies. Langmuir, Freundlich, and Temkin isotherm models are commonly utilized to analyze adsorption capacity and interaction types during the removal process [49,50]. The determination coefficient (R^2^) can be applied to Fig. 5 out the fitness of these models.

#### 3.9.1 Langmuir isotherm

The results from the investigation on Langmuir isotherm of the experimental data are as follows:

$$\frac{C_{e}}{q_{e}}=\frac{1}{K_{L}q_{m}}+\frac{C_{e}}{q_{m}}$$

Where $q_{e}$ (mg g^−1^) is the amount of adsorbed MPs at equilibrium, $q_{m}$ (mg g^−1^) is the maximum adsorption capacity; $C_{e}$ is the remaining concentration of MPs at equilibrium, and $K_{L}$ (L mg^−1^) is the Langmuir constant (Fig. 5a). The model indicates that the adsorbent surface is uniform/homogeneous, and the adsorption is performed as monolayer adsorption [51].

#### 3.9.2 Freundlich isotherm

The following equation is an illustration of the Freundlich isotherm model:

$$\ln\;q_{e}=\ln\;K_{F}+\frac{1}{n}\ln\;C_{e}$$

Where $q_{e}$ (mg g^−1^) is the amount of adsorbed MPs and $C_{e}$ is the remaining concentration of MPs at equilibrium; $K_{F}$ and n are the Freundlich constants for adsorption capacity and intensity, respectively (Fig. 5b). The experiment confirmed the existence of adsorbent with heterogeneous active binding sites, indicating multilayer adsorption, as confirmed by the compatibility with the Freundlich isotherm [52].

#### 3.9.3 Temkin isotherm

The Temkin isotherm model is a useful tool for estimating adsorbent-adsorbate surface interaction, as demonstrated in the analysis of experimental data [53].

$$q_{e}=\frac{RT}{b}\ln\left(A_{T}C_{e}\right)$$

$$q_{e}=BlnA_{T}+BlnC_{e}$$

The equations involve the Temkin isotherm constant (b), universal gas constant (RT), binding constant (A_T_), and adsorption heat constant (B), ensuring equilibrium adsorption energy (Fig. 5c). Table 2 displays the parameters obtained from fitting these isotherm models. In Fig. 5, the linear plots fitted to the (a) Langmuir, (b) Freundlich and (c) Temkin models are displayed where the correlation coefficients (R^2^) of these models were 0.98588, 0.90824, and 0.7697, respectively. The Langmuir model's calculation of PVC-MPs adsorption onto JSAC, with a capacity of 4.4668 mg/g, supports a monolayer adsorption process on homogenous material surface and the results are supported by the FTIR and SEM analysis. Based on these findings, the mechanism of PVC-MPs adsorption onto JSAC can be outlined as follows: (1) Electrostatic attractions between PVC-MPs and JSAC, (2) Hydrogen bonding interactions involving the oxygen-containing functional groups of JSAC and PVC-MPs, and (3) π−π interactions between JSAC and the aromatic ring of MPs. Consequently, the porous JSAC holds potential as an effective adsorbent for removing MPs from wastewater.

**Table 2:** Parameters derived from the isotherm model.

Langmuir Isotherm			Freundlich isotherm			Temkin isotherm		
q_max_	K_L_ (L/mg)	R^2^	K_F_ (L/mg)	n	R^2^	B_T_ (J/mol)	K_T_ (L/mg)	R^2^
0.446	227.118	0.985	31.617	2.12	0.908	−1.47	0.00066	0.769

### 3.10 Optimized geometry and interaction energy calculation of JSAC and PVC-MPs

In JSAC's fundamental composition, benzene rings form the core structure, a deduction drawn from solid-state ^13^C NMR experiments. The reaction models for the adsorption of microplastics impregnated in JSAC were established, centering on benzene ring clusters, aligning with the findings of Perry et al. [54]. To simulate the surface, zigzag edge sites were employed, given their proven suitability for JSAC modification. While the model size is not as crucial as the local shape of the active site, this study explored into a 7-fused ring to explore the impact of PVC-MPs coverage rates on adsorbent surfaces.

**Fig. 6:**
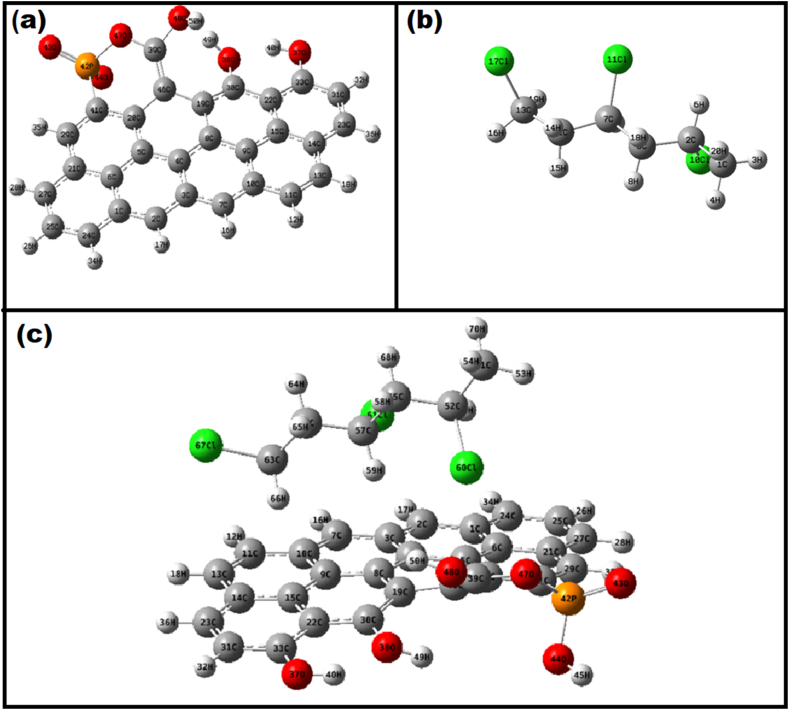
(a) Optimization structure of JSAC; (b) Optimization structure of PVC-MPs; (c) After adsorption of PVC-MPs surface on the JSAC.

The optimization of PVC adsorptions on JSAC was initiated through Gaussian 09 program package, employing the DFT-B3LYP/6-311+G (d, p) level of theory for the geometrical optimization of JSAC. Fig. 6 depicts the optimized structures of (a) JSAC; (b) PVC-MPs and (c) After adsorption of PVC-MPs surface on the JSAC. The optimized structure of the smallest modeled JSAC comprised 8 aromatic rings (Fig. 6a) and two repeating units of PVC-MPs (Fig. 6b). The complete basis set 6-31+G (d, p) was applied for other elements, with due consideration has been given for spin multiplicity to determine optimal structures. Theoretical parameters, encompassing frequency, density, and additional calculations, were computed using Multiwfn post-geometry optimization (Supplementary Table S2). Among these parameters, adsorption energy stands out as a crucial factor, offering insights into the adsorption behavior of PVC-MPs on JSAC and aiding in distinguishing between physical and chemical adsorption mechanisms (Fig. 6c). The adsorption energy (Eads) was calculated for the adsorbate on the substrate. This method proves instrumental in determining interaction energy values and conducting direct interaction energy calculations (Supplementary S3, Table S3) [37,55]. The interaction energy value after PVC-MPs adsorption is recorded as −269.003987 kcal/mol. This substantial difference aligns with experimental data, emphasizing the remarkable adsorption capacity of the adsorbent towards PVC-MPs.

### 3.11 Frontier molecular orbitals

**Fig. 7:**
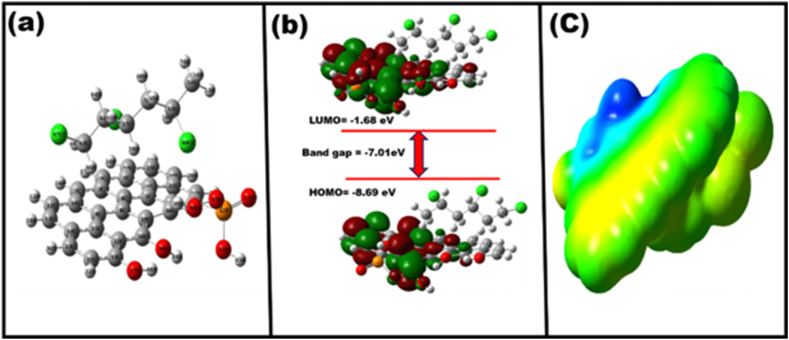
(a) Optimized geometry of JSAC-PVC-MPs; (b) HOMO and LUMO orbitals of JSAC-PVC-MPs and (c) electrostatic potential map are calculated DFT-B3LYP/6-311+G (d, p) level of theory.

Molecular orbital theory is a theoretical approach used to study the stability and reactivity of molecules. Within this framework, several quantities, such as the HOMO-LUMO gap (band gap) are calculated [56,57]. The frontier molecular orbitals, comprising the highest occupied molecular orbital (HOMO) and the lowest unoccupied molecular orbital (LUMO), play a crucial role in influencing the chemical stability [58]. Molecules that possess a significant difference in energy between their highest occupied molecular orbital (HOMO) and lowest unoccupied molecular orbital (LUMO), known as the frontier orbital gap, demonstrate limited chemical reactivity and strong kinetic stability. This is because it requires a considerable amount of energy to either add an electron to the energetically elevated LUMO or remove an electron from the energetically lowered HOMO. In this band gap analysis, a gap of −7.01 eV was found, indicating a strong interaction between JSAC and PVC (Supplementary Table S4). The topology of the HOMO and LUMO orbitals of the JSAC-PVC complex after the adsorption of PVC is depicted in Fig. 7b and a represents the optimized geometry of JSAC-PVC MPs. These figures demonstrate that the HOMO orbitals are localized on PVC, while the LUMO orbitals are localized on JSAC. The electrostatic potential map is demonstrated in Fig. 7c.

**Fig. 8:**
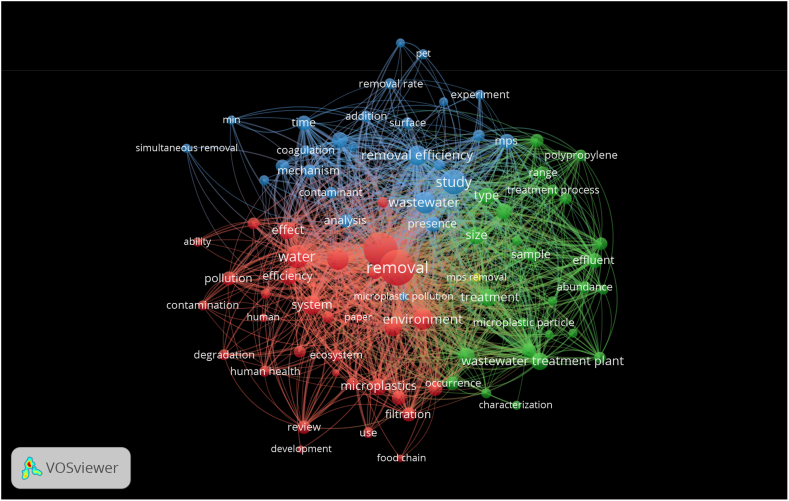
Scientometric analysis visualized the top 86 keywords from peer-reviewed articles published over the past ten years. A total of 4005 articles, identified on the Web of Science Core Collection using "removal of microplastics" as the search term, were analyzed. The analysis was conducted using the minimum co-occurrence frequency for all keywords, which were then graphically represented in VOSviewer in “Network visualization”, “overlay visualization (year)”, and “density visualization”. In these visualizations, each circle symbolizes a keyword, with its size indicating how frequently the keyword pairs have co-appeared in the literature. Different colors in the legend denote the average publication year of each keyword. (For interpretation of the references to color in this figure legend, the reader is referred to the Web version of this article.)

Here, Table 3 compares various current findings on MPs adsorption. The red area in Fig. 8 shows a strong emphasis on microplastics removal and its management. However, the comparative analysis from Table 3 clarifies that research with PVC-based MPs separation is less concerned. It is vividly clear from the comparison that coagulation and electrocoagulation are commonly used methods employed for separating MPs, but these approaches also face significant limitations. These drawbacks include the frequent need for electrode material replacement, elevated maintenance costs for electrodes, and adjustment of different factors such as electrode current density, electrode gap, electrolyte concentration, anodic material fouling, solution pH adjustment, and wastewater conductivity [59]. Consequently, introducing such a tertiary treatment on a commercial scale for MPs treatment proves to be quite challenging [22,60]. Furthermore, at an industrial level, chemical coagulation requires higher chemical concentrations and results in the generation of a substantial volume of sludge. Properly handling this hazardous sludge places additional financial and environmental burdens on both the economy and the ecosystem. On the other hand, sand filtration is another method of MPs removal with conventional treatment techniques deprived of advanced treatment. Compared with coagulation, the removal efficiency of MPs through sand filtration varies from 29 to 56 %. From comparison, it is also observed that adsorption is mostly considered for excellent MPs removal efficiency. Therefore, adsorption by JSAC has some advantages over other techniques, including simple setup and operation, strong adsorption capability for hydrophobic organic pollutants like PVC-MPs in aqueous environments, and no harmful by-products.

**Table 3:** Effectiveness of treatment methodologies for the removal of MPs.

Type of MPs	Methods	MP Size (μm)	Removal Efficiency (%)	Reference
PVC	Adsorption	250	94.1	This work
PS	Adsorption	1.45	92.0	[61]
PE, PET, PA	Adsorption	48	100.0	[30]
PVC	Adsorption	74	89	[62]
PS	Adsorption	>425	81	[63]
PS	Adsorption	6	48.5	[26]
Ps	Adsorption	5	61.7	[31]
PE	Adsorption	40–48	95.5	[25]
PET	Coagulation	100–400	54.7	[64]
PA, PP, PE, PVC	Active sludge	20–300	64.7	[65]
PS	Biodegradation	300–1250	43.7	[66]
PMMA, PE, CA, PP	Electrocoagulation	PE: 286.7	PE: 93.2	[67]
		PMMP: 6.3	PMMP: 91.7	
		PP: 1000-2000	PP: 98.4	
		CA: 1000-2000	CA: 98.2	
PE, PET	MBR	<5000	98.5	[68]
PE	Grit chamber	<5000	74.0	[69]
PS	Grit chamber	60–5000	66.0	[70]
PES, PE, PP	Dissolved air flotation	<5000	95.0	[71]
PET, PE, PP	Coagulation	5–10	44.5–75.0	[72]
PE	Coagulation	<500	8.3–61.2	[73]
PE	Coagulation	<500	11.6–90.9	[74]
PET, PE, PS	Sand filtration	<5000	29.0–41.0	[72]
PET, PA	Sand filtration	<5000	56.0	[75]
PE, PP, PA	Rapid sand filtration	<5000	74.0	[21]
PS	Biochar sand filtration	10	>95	[76]
PAC	Granular AC	<5000	56.8–60.9	[72]
PE, PP, PA	Ozone degrading	<5000	90.0	[21]

**Remarks:** PVC – polyvinyl chloride, PS – polystyrene, PE – polyethylene, PET - polyethylene terephthalate, PA- Polyamide, PP- Polypropylene, PMMA- Polymethyl methacrylate, CA- Cellulose Acetate, PES- polyethersulfone, PAC – polyaluminum chloride.

## 4 Conclusions

This work represents the first-time utilization of jute stick-activated (JSAC) charcoal as an eco-friendly adsorbent for extracting microplastics (MPs) from water. JSAC underwent a thorough characterization process that included UV–Vis spectroscopy, FT-IR, XRD, and SEM analysis. The purpose of these experiments was to verify the adsorbent qualities, efficient removal of PVC-MP particles, and evaluation of their morphological structure. JSAC achieved a maximum efficiency of 94.12 % during the experiment. This efficiency was seen at a pH of seven and within a time period of 120 min. The adsorbent dose used was 10 g.L-1. The mechanistic analysis reveals that the hydrophobic nature of microplastics and the presence of electron conjugation, electron interaction, and hydrogen bond interactions on the surfaces of PVC-MPs have an impact on the adsorption of PVC-MPs by JSAC.

Additionally, it is crucial to take into account the pH of the MPs solution, since it has a significant impact on regulating the surface charge of MPs. On the other hand, the amount of treatment agents and the length of time they are in contact with the water also have a substantial impact on the removal of MPs from water. This study examined the process of adsorption, specifically focusing on the kinetics and isotherm models. The pseudo-first-order kinetic equation and Langmuir isotherm models were shown to be suitable for explaining the physisorption of PVC-MPs onto JSAC. This process successfully removes PVC-MPs from aqueous environments. In addition, the significant adsorption of PVC-MP on JSAC was confirmed by DFT calculations, which showed an interaction energy of −269.00 kcal/mol. The results are in line with the experimental findings, and the comparison between the calculated and observed FTIR spectra shows a significant level of agreement with the experimental data. JSAC's observed characteristics indicate that it has the potential to be a cost-efficient and eco-friendly substance for removing MPs in aquatic environment.

## Funding

This work was supported by 10.13039/501100008804Ministry of Science & Technology of Bangladesh (Grant no.: special allocation-21-22-582 Phys).

## Data availability statement

Data sharing is not applicable to this article.

## CRediT authorship contribution statement

**Nur Alom:** Writing – original draft, Formal analysis, Data curation. **Tapati Roy:** Writing – original draft, Data curation. **Tanny Sarkar:** Methodology, Data curation, Conceptualization. **Md Rasel:** Writing – original draft, Formal analysis, Data curation. **Md Sanwar Hossain:** Writing – review & editing, Writing – original draft, Formal analysis. **Mamun Jamal:** Writing – review & editing, Writing – original draft, Supervision, Methodology, Funding acquisition, Formal analysis, Data curation, Conceptualization.

## Declaration of competing interest

The authors declare that they have no known competing financial interests or personal relationships that could have appeared to influence the work reported in this paper.
