# Supplementary material for: Removal of microplastics from aqueous media using activated jute stick charcoal
Source: Heliyon. 2024 Sep 3;10(18):e37380. doi: 10.1016/j.heliyon.2024.e37380 (PMC11414494; doi:10.1016/j.heliyon.2024.e37380)
Supplement: Multimedia component 1 [file mmc1.docx]

**Removal of microplastics from aqueous media using activated jute stick charcoal**

Nur Alom^a,c^, Tapati Roy^b,c^, Tanny Sarkar^a,c^, Md Rasel^a,c^, Md Sanwar Hossain^a,c^, Mamun Jamal^a,c*^

^a^Department of Chemistry, Khulna University of Engineering & Technology, Khulna 9203, Bangladesh

^b^Department of Agronomy, Faculty of Agriculture, Khulna Agricultural University, Khulna, Bangladesh

^c^Microplastics Solution Ltd., Incubation Centre, KUET Business Park, Khulna, Bangladesh

^*^Corresponding authors Email: [mamun.jamal@chem.kuet.ac.bd](mailto:mamun.jamal@chem.kuet.ac.bd)

**S1. Analysis of charcoal**

For proximate analysis, to determine moisture, volatile, ash, and fixed carbon [1].

**Table S1**

Composition of charcoal powder from jute stick.

| Heat  °C | Moisture  % | Volatile  % | Ash  % | Fixed Carbon  % |
| --- | --- | --- | --- | --- |
| 500 | 10 | 44.4 | 1.865 | 43.685 |
| 400 | 7 | 45.8 | 1.234 | 42.293 |
| 300 | 12 | 50.0 | 0.707 | 40.936 |

**S2. SEM**

Figure S1 shows the SEM images of (a) jute sticks powder, (b) jute sticks charcoal (JSC), (c) jute sticks activated charcoal (JSAC); (d-e) JSAC after adsorption. The images represent the morphological changes of the carbon materials during pyrolysis, activation, and adsorption. The jute stick surfaces are relatively smooth solid surfaces (Fig. S1a) with long ridges, resembling a series of parallel lines as observed in other biomasses [2]. When jute sticks were subjected to pyrolysis to produce charcoal, most of the organic volatiles evolved, leaving behind the ruptured surface of charcoal with a small number of pores (Fig. S1b) [3]. Fig. S1(c) shows the activation of charcoal with HCl which represents a number of different sizes of pores with increased surface area [3-4]. The number of pores is increased in case of JSAC compared to JSC. This may happen due to lignin dissolution from jute sticks through the activity of HCl. Moreover, it is vividly clear from Fig. S1(d) and (e), there is a reduction in the pore size of the JSAC after adsorption of MPs compared to JSC and JSAC. This was possibly due to the adsorption properties JSAC which revealed the adsorption of the large number of PVC-MPs.

The scanning electron microscopy (SEM) settings used for these observations, including magnification, spot size, working distance, and high voltage (mag 1411x, spot 5.0, W 9.9 mm, HFW 160 µm, HV 20.0 kV for Figure 1(d); mag 960x, spot 5.0, W 10.0 mm, HFW 155 µm, HV 20.0 kV for Figure 1(e), ensure detailed visualization of these structural changes.

SEM studies were performed on finely ground jute stick powder using a JEOL JCM-7000, Benchtop scanning electron microscope. The jute sticks were processed into powder form to ensure a uniform surface for analysis. The SEM imaging was conducted under high vacuum (HV) mode with an accelerating voltage of 15.0kV and a working distance (WD) of 11.9 mm. The magnification used for capturing the images was 1000x, with a field of view of 128.0 µm by 96.0 µm. Prior to imaging, the jute stick powder samples were coated with a thin layer of gold to improve conductivity and enhance image clarity. This preparation allowed us to observe the surface morphology and structural characteristics of the JS powder, JSC, JSAC, and JSAC after adsorption in detail.


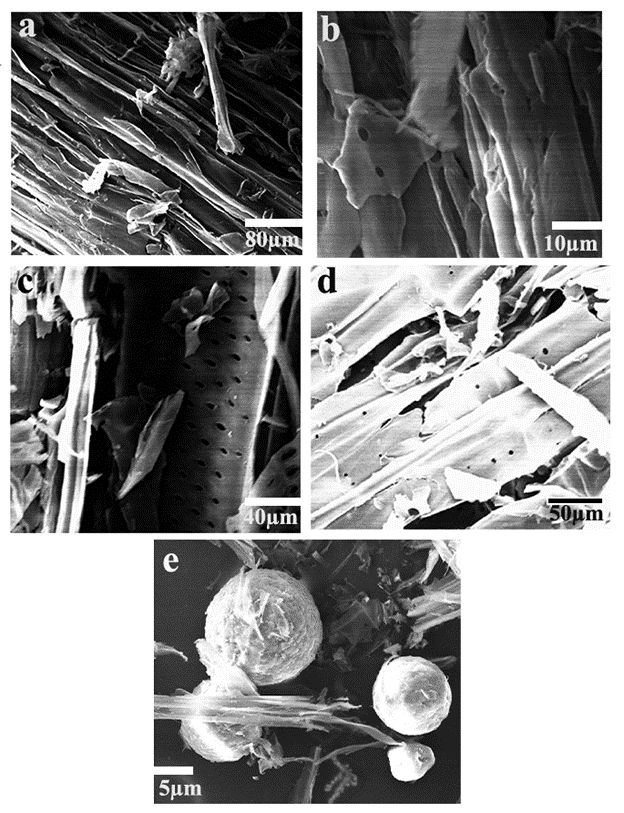


**Fig. S1.** Scanning electron micrograph of (a) jute sticks; (b) charcoal; (c) JSAC; (d-e) JSAC after adsorption at different magnifications.

**Table S2**

Energy and dipole moment of JSAC and PVC-MPs.

| **Species** | **Energy (Hartree)** | **Dipole moment (Debye)** |
| --- | --- | --- |
| JSC(AC) | -1906.3794 | 2.8452 |
| PVC | -1615.8945 | 3.3635 |
| JSC(AC)-PVC | -3522.70259 | 4.3311 |

The energies of JSAC, both before and after PVC-MPs adsorption, were computed at the same theoretical level, as presented in Table S2, which details the calculated simple interaction energies.

**S3. Vibrational studies**

The infrared (IR) spectra of the studied compounds were computed at the DFT/6-311G+ (2d, p) level of theory and scaled by 0.9688 [5]. The comparison between calculated and observed FTIR spectra, detailed in Table S3, demonstrates good agreement. This concordance lends confidence to a more reliable assignment of IR active modes of vibration by juxtaposing experimental FT-IR intensities with the computed wavenumbers. The discussion herein focuses solely on the JSAC and JSAC-PVC-MPs systems, with the exclusion of larger systems to avoid unnecessary complexity.

**Table S3**

Provides a detailed comparison between experimental FTIR spectra and calculated (scaled) IR spectra at the DFT B3LYP/6-311G+ (d, p) level of theory.

| Cal./IR(cm^-1^) JSC | Exp./FT-IR(cm^-1^)  JSC | Cal./IR(cm^-1^) PVC | Cal./IR(cm^-1^)  JSC-PVC | Exp./Ft-IR(cm^-1^) JSC-PVC |
| --- | --- | --- | --- | --- |
| 655 | 619 | 591 | 685 | 615, 640 |
| 774 | 781 |  | 824 | 763 |
| 860 | 833 | 846 | 896 | 824, |
| 891 | 902 | 960 | 962 | 902 |
| 913 | 960 | 1006 | 1035 | 1035 |
| 1000, 1053 | 1043 |  | 1020 | 1035 |
| 1105 | 1110 | 1184,1143 | 1161, 1191 | - |
| 1250,1205 | 1260 | 1281 | 1262 | 1255, 1273 |
| 1313,1320,  1337 | 1338 | 1354 | 1338, 1322 | 1336 |
| 1436, 1498 | 1425 | 1423 |  | 1402,1429 |
| 1552 | 1573 | 1503 | 1591 | 1608 |
| 1609,1619  1648 | 1610 | - | - | 1697 |
| 3217 | 3294 | 3141 | - | 3188-3700 |
| 3525,3731 | 3543 | 3044 | - | 3550 |
| 3731 | 3745 | 3141 | - | 3745 |
| 3809 | 3805 | - | - | - |

In the vibrational spectra of JSAC-PVC MPs after PVC-MPS adsorption, the emergence of additional peaks is observed, absent in the JSAC spectrum. Furthermore, the adsorption of PVC-MPs not only introduces new signals but also induces significant shifts in the positions of some existing peaks. The supplementary peaks signify interactions between PVC-MPs and JSAC. All pertinent observations regarding additional peaks and shifts in existing peaks are compiled in Table S3.

**Table S4**

Values of the HOMO, LUMO energy (eV), and HOMO-LUMO energy gap (band gap) of JSAC-PVC are calculated at B3LYP/6-311G.

| Name | HOMO (eV) | LUMO (eV) | Band gap (eV) |
| --- | --- | --- | --- |
| JASC-PVC | -8.69 | -1.68 | -7.01 |

**Reference:**

[1] M. N. Islam *et al.*, "Synthesis and characterization of activated carbon prepared from jute stick charcoal for industrial uses," *Scholars International Journal of Chemistry and Material Sciences,* vol. 5, no. 3, pp. 33-39, 2022.

[2] B. P. Kumar, K. Shivakamy, L. R. Miranda, and M. Velan, "Preparation of steam activated carbon from rubberwood sawdust (Hevea brasiliensis) and its adsorption kinetics," *Journal of Hazardous Materials,* vol. 136, no. 3, pp. 922-929, 2006.

[3] M. Asadullah, M. Asaduzzaman, M. S. Kabir, M. G. Mostofa, and T. Miyazawa, "Chemical and structural evaluation of activated carbon prepared from jute sticks for Brilliant Green dye removal from aqueous solution," *Journal of Hazardous Materials,* vol. 174, no. 1-3, pp. 437-443, 2010.

[4] M. A. Aziz, I. R. Chowdhury, M. A. J. Mazumder, and S. Chowdhury, "Highly porous carboxylated activated carbon from jute stick for removal of Pb ^2+^ from aqueous solution," *Environmental Science and Pollution Research,* vol. 26, pp. 22656-22669, 2019.

[5] C. Copeland, O. Menon, D. Majumdar, S. Roszak, and J. Leszczynski, "Understanding the influence of low-frequency vibrations on the hydrogen bonds of acetic acid and acetamide dimers," *Physical Chemistry Chemical Physics,* vol. 19, no. 36, pp. 24866-24878, 2017.
